# Muscle synergies and metabolic adaptations during perturbed walking in older adults

**DOI:** 10.1038/s41598-025-07835-4

**Published:** 2025-07-02

**Authors:** Samuel D’Emanuele, Marco Ghislieri, Laura Ghiotto, Francesca Morra, Lorenzo Budel, Doriana Rudi, Simone Bettega, Gennaro Boccia, Federico Schena, Cantor Tarperi

**Affiliations:** 1 Department of Neurosciences, Biomedicine and Movement Sciences, University of Verona, Verona, Italy; 2 PolitoBIOMed Lab and Department of Electronics and Telecommunications, Polytechnic University of Turin, Turin, Italy; 3 Department of Clinical and Biological Sciences, University of Turin, Turin, Italy; 4 CeRiSM (Research Center Sport Mountain and Health), University of Verona and Trento, Rovereto, Italy

**Keywords:** Locomotion, Motor modules, Neuromuscular control, Energetic cost, Motor neuron, Motor control, Central pattern generators

## Abstract

Walking is an accessible and beneficial form of physical activity for older adults. However, when performed under perturbed conditions, it can place greater demands on both neuromuscular control and metabolic efficiency, highlighting the need to better understand the underlying adaptations. We compared stable and perturbed walking on a treadmill at three different speeds in 16 healthy older adults. Muscle synergies were extracted from electromyographic signals of eight lower limb muscles, oxygen uptake (VO_2_), minute ventilation, heart rate, and rate of perceived exertion (RPE) were also collected. Perturbed walking at 3 km/h increased oxygen uptake (*p* = 0.003; *d *= -0.52) and energetic cost (EC; *p* = 0.003; *d = *-1.12) compared to the stable condition. VO_2_ (*p* < 0.001; $\:{\eta\:}_{p}^{2}$ = 0.92), EC (*p = *0.02; $\:{\eta\:}_{p}^{2}$ = 0.25) and RPE (χ^2^ = 4.45; *p* = 0.04) were higher at 3 km/h than at higher speeds. Four muscle synergies were required to accurately model the neuromuscular control during both conditions. Full-width at half maximum revealed that the transition from stable to perturbed walking led to statistically significant modifications in gait synergy composition (*p =* 0.01; $\:{\eta\:}_{p}^{2}$= 0.04). Weight sparsity revealed no significant differences between conditions. These results suggest that the transition from an accurate to a more robust locomotor control for managing continuous perturbations may come at the expense of increased metabolic demand.

**Supplementary Information:**

The online version contains supplementary material available at 10.1038/s41598-025-07835-4.

## Introduction

Regular physical activity is one of the keys to successful aging. As recommended by the World Health Organization, older adults (> 65 years) should engage in at least 150–300 min of moderate-intensity aerobic physical activity, 75–150 min of vigorous-intensity aerobic physical activity, or an equivalent combination of moderate- and vigorous-intensity activity per week to reap substantial health benefits^1^. Among the various physical and sports activities that can be proposed to achieve this goal, walking is a low-cost option that most older people^2^ can do with health benefits at various levels^3–6^.

Although walking in places such as shopping malls is commonly considered safe and comfortable^7,8 ^moving over uneven terrain is a daily challenge for older adults because of the need for the Central Nervous System (CNS) to flexibly modify its control strategies to cope with varying surface conditions. These demands have an impact not only from a cognitive perspective but also from a metabolic perspective. The Energy Cost (EC) of locomotion (amount of metabolic energy required to move a given distance, typically expressed as J/kg/m) increases on irregular surfaces (e.g., sand, snow, and trails) compared to smooth surfaces (e.g., tarmac, flooring, and track)^9–15 ^but the underlying biomechanical and neuromuscular mechanisms are not fully understood. Among the various parameters that may affect the EC between uneven and smooth terrains, one must consider step width and step length, as both tend to change with age^16^. Irregular terrains may also perturb gait step-by-step and cause greater variability. Such surfaces might also demand more mechanical work from the legs, regardless of their effects on step parameters. Kuo^17^ previously hypothesized that the walking economy improves by pushing with the rear leg just before the collision with the front leg. This push redirects the body’s center of mass and, if timed correctly, can reduce the amount of negative work done during the collision. Irregular terrains may alter the timing of these events so that a collision occurring earlier or later than the push would lead to increased negative mechanical work. This would then require muscles to compensate and perform more positive work elsewhere, as stable walking requires overall zero net work. It is difficult to predict how work will be distributed among the lower limb joints, but altered timing would likely require more work overall and, thus, a higher metabolic energy expenditure.

Another potential factor contributing to increased energy expenditure could be the co-activation of muscles that may occur in unpredictable or irregular terrain conditions or when there is a potential risk of falling. Indeed, on uneven surfaces, healthy adults adopt several gait modifications, such as a lowered center of mass, greater co-contractions during stance, and increased toe clearance during swing phase^18^. Supporting this, a study by Voloshina and colleagues^13^ found that walking on uneven terrain with a surface variability of only 2.5 cm increased net metabolic cost by 28%, similar to the effort required for walking up a 2% incline^19^. Despite only modest changes in step strategy being demonstrated, with a 4% decrease in step length and no significant change in step width, variability in both step length and width increased by 22% and 36%, respectively. This increased variability, along with a more crouched posture and greater muscle activity (especially at the hip), likely contributed to the metabolic cost. Biomechanically, the hip performed 62% more positive work, and the knee 26% more negative work. Although they observed an increase in vastus lateralis and vastus medialis muscle activities during the stance phase, it is not possible to estimate the cost of co-activations. However, these co-activations likely contributed to the increased metabolic cost on uneven terrains. In another study by Hawkins et al.^18, ^participants increased limb work by up to 20% on uneven terrain^20 ^with older adults relying more on proximal muscles at the hip and knee, which are more susceptible to age-related decline. Interestingly, this increase in proximal workload was partially offset by reduced ankle kinetics.

From this point of view, new methodological approaches are needed to evaluate motor control strategies during gait over different terrains in a quantitative and non-invasive way. One promising method involves analyzing muscle synergies^21^—low-dimensional control modules through which the CNS coordinates multiple muscles using a limited set of time-dependent neural commands^22,23^. These synergies generate complex electromyographic activity via descending or afferent pathways^24,25^ facilitating motor control (e.g., locomotor control) with some degree of supraspinal regulation^26^. These synergies are extracted from surface EMG recordings using algorithms such as Non-Negative Matrix Factorization (NNMF)^27^. Research shows that human locomotion^28,29 ^in both healthy and pathological conditions^30,31 ^can be described using just 4 to 5 synergies^32,33 ^depending on factors like the muscle set, preprocessing, and algorithm used. Remarkably, these synergies tend to be preserved even under continuous external perturbations, suggesting that the CNS compensates by expanding and modulating basic motor commands to maintain stable movement serves as a compensatory strategy to deal with the perturbations^34,35^.

Thus, muscle synergies enable an objective and non-invasive assessment of the CNS’s motor control strategies during different tasks. In this context, muscle synergies and the analysis of EC are promising frameworks for evaluating shifts in neuromuscular and metabolic strategies in response to different conditions, such as walking on even and uneven terrains. Moreover, integrating metabolic data with muscle synergy analysis may offer valuable insights into the central correlates of muscle synergies, further elucidating the neural mechanisms underlying motor control adaptations.

This study aims to address a knowledge gap regarding the metabolic and neuromuscular adaptations required for older adults to walk successfully during unpredictable medio-lateral variations, a common challenge encountered in daily life (such as normal walking on uneven terrain, as typically found in extra-urban, rural, or mountainous areas). Accordingly with previous literature, we hypothesized that, compared to flat walking, walking on a perturbed surface at the same speed would require adaptations in motor control strategies—shifting from accurate (i.e., mature and functionally fine-tuned) to more robust ones (i.e., better suited to handle unexpected errors)^35^—along with an increase in energy cost (EC)^13 ^and no change in perceived effort^36^. Although the advantages of physical activity for aging populations are well documented, there is a paucity of research examining the specific EC and motor control strategies employed when walking over irregular surfaces or during unpredictable stimuli. By examining the interplay between motor control strategies and energy expenditure, this study provides novel insights into the impact of unpredictable perturbations on gait performance in healthy older adults. Notably, while numerous studies have independently investigated the effects of various terrain conditions on the metabolic and neuromuscular systems, this is the first study to simultaneously explore both metabolic and neuromuscular control changes resulting from medio-lateral perturbations. These findings may potentially inform strategies for improving mobility, reducing fall risk, and promoting overall health in older adults, thereby enhancing the effectiveness of physical activity interventions targeted at this population.

## Results

The metabolic and neuromuscular performance of older adults walking during Flat Condition (FC) and Unpredictable Roll Variations (URV) conditions were compared using metabolic parameters including Energy Cost (EC, metabolic energy required per unit distance), Oxygen Consumption (VO₂, volume of oxygen consumed), Heart Rate (HR), and Minute Ventilation (VE, volume of air breathed per minute), as well as neuromuscular metrics such as muscle synergy composition (optimal number of muscle synergies and description of the activation coefficients and weight vectors), Variance Accounted For (VAF, goodness of muscle synergy model in reconstructing the original EMG data), Full-Width at Half Maximum (FWHM, number of activation coefficient time-instants exceeding half of the signal’s maximum value), Weight Sparsity (WS, number of muscles mainly enrolled within each muscle synergies), and Cosine Similarity of weight vectors (CS, degree of correlation between couple of weight vectors extracted during walking conditions). Moreover, the Rated Perceived Exertion (RPE, subjective measure of effort) data were analyzed during both walking conditions.

### Metabolic analysis

The results of the metabolic analysis are detailed in Table 1, with the indication of the statistically significant differences between walking conditions at the same speed (indicated through asterisks). To notice, given that at moderate exercise intensities a steady state is generally reached within the first three minutes, to identify potential duration-dependent metabolic alterations that could arise due to prolonged exposure, we conducted an additional sub-analysis of the physiological variables at the midpoint of each stage (i.e., EC_3min_ and EC_6min_).

**Table 1:** Metabolic and cardiopulmonary parameters were measured at different walking speeds (3, 4, and 5 km/h) and conditions (Flat = FC; Unpredictable Roll Variations = URV). The table reports energy cost assessed in two different time windows (EC_3min_ and EC_6min_), oxygen consumption (VO₂), heart rate (HR), and Minute Ventilation (VE) as mean ± standard deviation. *indicates statistical significance (*p ≤ 0.05.) between two conditions at the same speed.

Metabolic and cardiopulmonary parameters		Walking Speed		
		3 km/h	4 km/h	5 km/h
EC_3min_ [J/kg/m]	FC	2.80 ± 0.76*	3.01 ± 0.81	3.24 ± 0.40
	URV	3.53 ± 0.97*	3.04 ± 0.79	3.23 ± 0.57
EC_6min_ [J/kg/m]	FC	2.61 ± 0.77*	2.87 ± 0.44	3.13 ± 0.45
	URV	3.33 ± 0.65*	3.0 ± 0.52	3.22 ± 0.47
VO_2_ [ml/min]	FC	749 ± 143*	951 ± 185	1153 ± 216
	URV	887 ± 232*	986 ± 210	1180 ± 246
HR [bpm]	FC	86 ± 13	93 ± 13	101 ± 12
	URV	87 ± 11	95 ± 13	101 ± 12
VE [L/min]	FC	24.39 ± 5.32	29.46 ± 6.16	35.33 ± 8.05
	URV	26.30 ± 6.01	30.13 ± 5.66	36.41 ± 7.54

The Shapiro-Wilk test showed that all the metabolic data were normally distributed.

2-way ANOVA revealed no statistically significant difference in EC_3min_ values between flat and unpredictable roll variations conditions based on Speed ($\:F$(2, 26) = 1.74, *p* = 0.20; $\:{\eta\:}_{p}^{2}$ = 0.12). Significant differences were detected based on Condition ($\:F$(1, 13) = 9.08; *p *= 0.01; $\:{\eta\:}_{p}^{2}$ = 0.41) and Speed × Condition ($\:F$(2, 26) = 11.1; *p* < 0.001; $\:{\eta\:}_{p}^{2}$ = 0.46). *Post-hoc* analysis revealed a significant interaction effect between flat and perturbed conditions at 3 km/h (*p*_*bonf *_= 0.02; Cohen’s *d *= -1.11). Considering EC_6min_, statistically significant differences were detected between flat and perturbed conditions based on Speed ($\:F$(2, 26) = 4.33, *p* = 0.02; $\:{\eta\:}_{p}^{2}$ = 0.25). Significant differences were detected based on Condition ($\:F$(1, 13) = 14.97; *p *= 0.002; $\:{\eta\:}_{p}^{2}$ = 0.54) and Speed × Condition ($\:F$(2, 26) =13.49; *p* < 0.001; $\:{\eta\:}_{p}^{2}$ = 0.51). *Post-hoc* analysis revealed a significant interaction effect between flat and perturbed conditions at 3 km/h (*p*_*bonf *_= 0.003; Cohen’s *d *= -1.12).

2-way ANOVA revealed a statistically significant difference in VO_2_ values between flat and perturbed conditions based on Speed ($\:F$(2, 28) = 158.1, *p* < 0.001; $\:{\eta\:}_{p}^{2}$ = 0.92), Condition ($\:F$(1, 14) = 11.06; *p *= 0.005; $\:{\eta\:}_{p}^{2}$ = 0.44), and Speed × Condition ($\:F$(2, 28) = 8.08; *p* < 0.002; $\:{\eta\:}_{p}^{2}$ = 0.37). *Post-hoc* analysis revealed an interaction between flat and perturbed conditions at 3 km/h (*p*_*bonf *_= 0.003; Cohen’s *d *= -0.52).

2-way ANOVA revealed a statistically significant difference in HR values based on Speed ($\:F$(1.393, 28) = 73.63, *p* < 0.001; $\:{\eta\:}_{p}^{2}$ = 0.84). No differences were detected for Condition ($\:F$(1, 14) = 3.35; *p *= 0.09; $\:{\eta\:}_{p}^{2}$ = 0.19) and Speed × Condition ($\:F$(2, 28) = 0.95; *p* = 0.39; $\:{\eta\:}_{p}^{2}$ = 0.06). The *post-hoc* analysis did not reveal any interaction effect between the conditions matched for speed.

Finally, no significant differences in VE for Speed ($\:F$(2, 30) = 95.71; *p* < 0.001; $\:{\eta\:}_{p}^{2}$ = 0.87), Condition ($\:F$(1, 15) = 6.65; *p *= 0.02; $\:{\eta\:}_{p}^{2}$ = 0.31), and Speed × Condition ($\:F$(2, 30) = 0.85; *p* = 0.44; $\:{\eta\:}_{p}^{2}$ = 0.05) were detected.

The raincloud plots of the metabolic parameters are represented in Fig. 1S in the Supplementary Materials.

### Muscle synergy composition

**Fig. 1:**
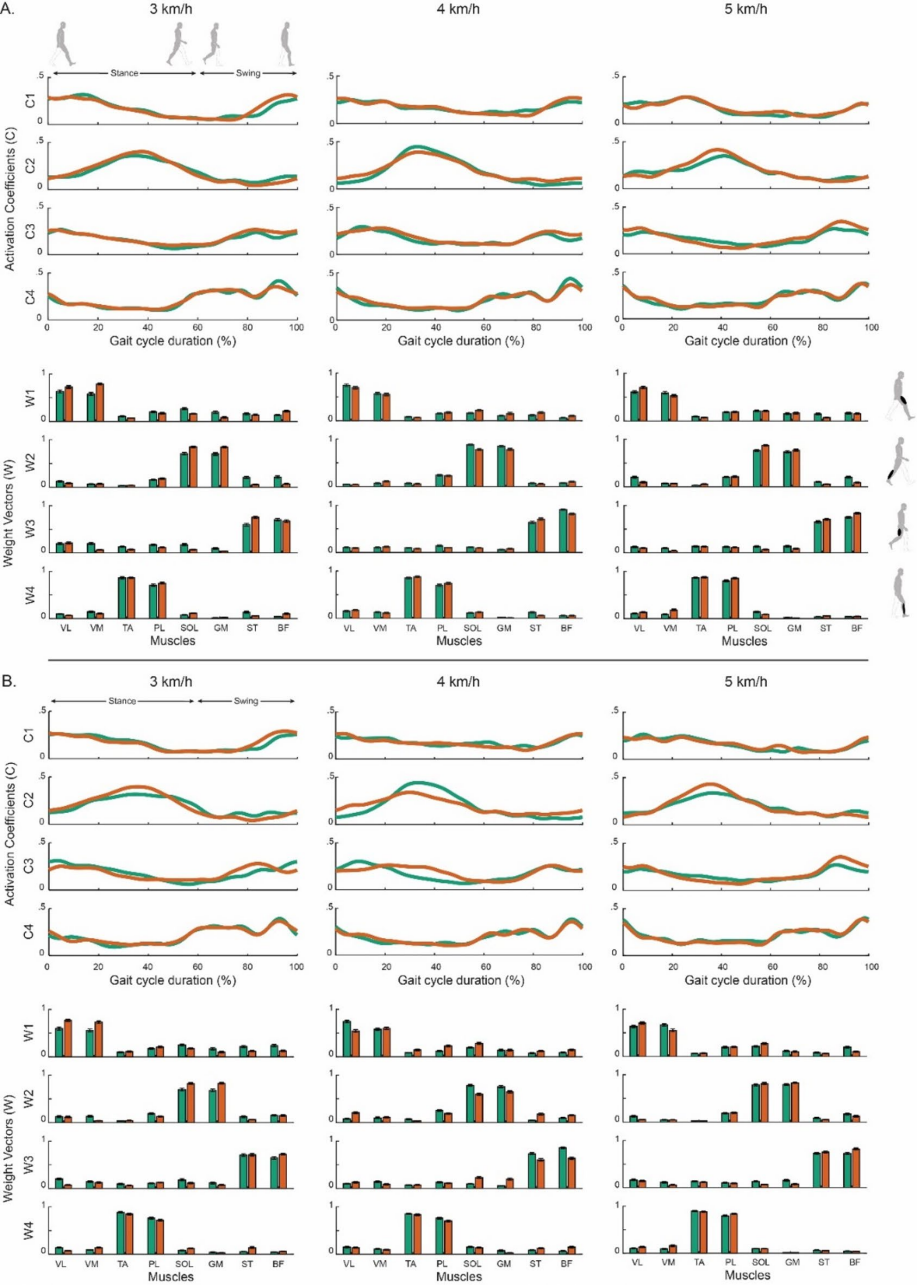
Comparison of muscle synergies averaged across the sample population during stable (green) and perturbed (orange) walking conditions. (**A**) presents the muscle synergies extracted during the first three minutes of each walking condition, while (**B**) displays those from the final three minutes. Activation coefficients (C) and weight vectors (W) are represented as mean ± standard errors. Muscle abbreviations: VL – vastus lateralis, VM – vastus medialis, TA – tibialis anterior, PL – peroneus longus, SOL – soleus, GM – gastrocnemius medialis, ST – semitendinosus, and BF – biceps femoris.

Figure 1 shows the average weight vectors and activation coefficients of the muscle synergies identified during the two walking conditions (flat and unpredictable roll variations) at different speeds (i.e., 3, 4, and 5 km/h).

On average, four muscle synergies were required to accurately model the original EMG signals across both walking conditions and all walking speeds. From a biomechanical standpoint, each muscle synergy can be linked to a distinct motor function, as outlined below:

- First muscle synergy is crucial for hip stabilization during heel strike and the load acceptance phases, primarily driven by the activation of the VL and VM muscles;
- Second muscle synergy is responsible for propulsion during the mid and late stance phases, primarily characterized by the activation of the SOL and GM muscles;
- Third muscle synergy contributes to leg and foot deceleration during the late swing phase, preparing the limb for foot contact. This synergy is mainly characterized by the activation of the ST and BF muscles;
- Fourth muscle synergy ensures forefoot clearance during the swing phase, primarily driven by the activation of the TA and PL muscles.

The composition of muscle synergies (i.e., activation coefficients and weight vectors) remains largely consistent across both walking conditions and all walking speeds, suggesting a well-organized and adaptable control system that effectively accommodates varying walking conditions.

### Muscle synergy analysis

The average muscle synergy-derived values are detailed in Tables 2 , 3, and Table 4 with the indication of the statistically significant differences between walking conditions and speeds (indicated through asterisks). To identify potential duration-dependent alterations in motor control resulting from prolonged exposure, we analyzed muscle synergy-derived parameters across two consecutive 3-minute intervals (i.e., I Half and II Half).

**Table 2:** Variance accounted for (VAF) and Full-Width at half maximum (FWHM) values for each condition (Flat = FC; Unpredictable Roll Variations = URV) and speed divided in the two 3-minute time-windows (I half vs. II half). Parameters’ values are reported as mean ± standard error over the sample population. Asterisks (*), double asterisks (**), and dagger (^†^) represent statistically significant differences between flat and perturbed conditions.

Muscle synergy-derived parameters			Walking speed		
			3 km/h	4 km/h	5 km/h
VAF [%]	I Half	FC	93.9 ± 1.7	93.4 ± 2.2	93.0 ± 3.1
		URV	93.5 ± 1.8	92.2 ± 2.2	93.0 ± 1.7
	II Half	FC	92.9 ± 1.9	92.6 ± 1.8	93.1 ± 1.9
		URV	92.9 ± 1.9	92.3 ± 1.9	92.9 ± 1.2
FWHM [%GC]	I Half	FC	24.3 ± 4.1*	22.4 ± 6.3**	23.5 ± 5.4
		URV	28.1 ± 7.2*	27.2 ± 5.6**	24.6 ± 5.2
	II Half	FC	**23.2 ± 4.9** ^**†**^	24.3 ± 8.9	22.6 ± 5.6
		URV	**26.1 ± 4.2** ^**†**^	25.9 ± 6.3	23.3 ± 7.5

**Table 3:** Weight sparsity (WS) values for each muscle synergy (from syn 1 to syn 4) and each walking speed. Parameters’ values are reported as mean ± standard error over the sample population. Flat = FC; Unpredictable Roll Variations = URV.

Weight sparsity		Walking speed											
		3 km/h				4 km/h				5 km/h			
		Syn 1	Syn 2	Syn 3	Syn 4	Syn 1	Syn 2	Syn 3	Syn 4	Syn 1	Syn 2	Syn 3	Syn 4
I Half	FC	3.6 ± 1.2	3.4 ± 1.2	3.9 ± 1.6	3.3 ± 1.2	4.1 ± 1.2	3.8 ± 1.2	3.8 ± 1.4	4.0 ± 1.2	4.1 ± 1.1	3.8 ± 1.2	3.8 ± 1.3	3.3 ± 1.7
	URV	4.0 ± 1.5	3.7 ± 1.3	4.1 ± 1.4	3.6 ± 1.6	3.8 ± 1.3	3.6 ± 0.8	3.8 ± 1.3	3.6 ± 1.5	3.8 ± 1.2	3.8 ± 1.1	3.8 ± 0.7	4.1 ± 1.4
II Half	FC	3.8 ± 1.0	3.9 ± 1.2	3.6 ± 1.2	3.6 ± 1.5	3.6 ± 1.2	3.3 ± 1.2	3.4 ± 1.5	3.3 ± 1.1	4.2 ± 0.8	3.8 ± 1.3	4.0 ± 1.1	3.9 ± 1.5
	URV	4.3 ± 1.4	3.9 ± 1.5	3.2 ± 1.4	3.9 ± 1.2	3.8 ± 1.1	3.6 ± 1.3	3.4 ± 1.4	3.8 ± 0.9	4.5 ± 1.1	3.9 ± 1.3	3.4 ± 1.6	3.7 ± 1.3

**Table 4:** Cosine similarity (CS) values between walking conditions for each muscle synergy (from Syn 1 to Syn 4) and each walking speed. Parameters’ values are reported as mean ± standard error over the sample population.

Cosine similarity	Walking speed											
	3 km/h				4 km/h				5 km/h			
	Syn 1	Syn 2	Syn 3	Syn 4	Syn 1	Syn 2	Syn 3	Syn 4	Syn 1	Syn 2	Syn 3	Syn 4
I Half	0.66 ± 0.09	0.78 ± 0.09	0.73 ± 0.07	0.87 ± 0.07	0.85 ± 0.04	0.83 ± 0.08	0.81 ± 0.07	0.85 ± 0.07	0.84 ± 0.06	0.86 ± 0.08	0.78 ± 0.06	0.94 ± 0.03
II Half	0.68 ± 0.06	0.81 ± 0.08	0.74 ± 0.08	0.86 ± 0.06	0.76 ± 0.08	0.69 ± 0.09	0.71 ± 0.09	0.87 ± 0.06	0.84 ± 0.06	0.93 ± 0.06	0.85 ± 0.06	0.93 ± 0.03

The Shapiro-Wilk test showed that all the muscle synergy data were normally distributed.

3-way ANOVA revealed no statistically significant differences in VAF values based on Speed ($\:F$(2, 178) = 1.91, *p* = 0.15; $\:{\eta\:}_{p}^{2}$ = 0.02), Condition ($\:F$(1, 178) = 0.37, *p *= 0.54; $\:{\eta\:}_{p}^{2}$ = 0.002), and Time Window ($\:F$(1, 178) = 1.27, *p* = 0.26; $\:{\eta\:}_{p}^{2}$ = 0.007). The *post-hoc* analysis did not reveal any interaction effect between factors.

No statistically significant differences in FWHM values based on Speed ($\:F$(2, 178) = 2.36, *p* = 0.10; $\:{\eta\:}_{p}^{2}$ = 0.03) and Time Window ($\:F$(1, 178) = 0.42, *p *= 0.52; $\:{\eta\:}_{p}^{2}$ = 0.002) were detected. However, a statistically significant difference was detected based on Condition ($\:F$(1, 178) = 6.80, *p* = 0.01; $\:{\eta\:}_{p}^{2}$= 0.04). The *post-hoc* analysis did not reveal any interaction effect between factors.

3-way ANOVA revealed no statistically significant differences in WS values based on Condition, Time Window, and Speed.

Finally, 2-way ANOVA revealed no statistically significant differences in CS values based on Time Window and Speed.

The raincloud plots of the muscle synergy-derived parameters are represented in Fig. 2S in the Supplementary Materials.

### Exertion analysis

The Rated Perceived Exertion (RPE) data analyzed using the Shapiro-Wilk test revealed a deviation from normal distribution. Consequently, the Friedman test was conducted to assess the differences between the two conditions. A significant difference was found (see Table 5) at 3 km/h (χ^2^(1, *N* = 16) = 4.45; *p* = 0.04) and at 5 km/h (χ^2^(1, *N* = 16) = 6.23; *p* = 0.01), while the statistical significance was not reached for 4 km/h (χ^2^(1, *N* = 16) = 1.92; *p* = 0.17).

**Table 5:** Rated perceived exertion (RPE) for both conditions at each walking speed. *indicates statistical significance (*p ≤ 0.05) between two conditions at the same speed.

Exertion parameter		Walking speed		
		3 km/h	4 km/h	5 km/h
RPE	FC	6.6 ± 3.4*	15.2 ± 8.7	23.9 ± 9.1*
	URV	10.4 ± 7.2*	17.1 ± 9.2	27.7 ± 11.9*

## Discussions

The present study examined the metabolic and neuromuscular control changes during a walk at three different speeds (3, 4, and 5 km/h) in two conditions: stable/flat and unpredictable/perturbed in healthy older adults. The aim was to analyze modular locomotor control through muscle synergy, cardio-pulmonary, and perceived exertion measures. It was hypothesized that perturbed walking at the same speed would result in increased neuromuscular and metabolic demands, reflected by a shift from accurate (i.e., mature, functionally fine-tuned) to more robust (i.e., able to cope with unexpected errors) motor control, higher energy cost (EC), and unchanged perceived effort.

Our results showed that participants had higher VO_2_ values and, consequently, higher EC_6min_ in the perturbed condition at 3 km/h compared to the flat condition (VO_2_ = + 18%; EC_6min_ = + 28%). In addition, VO_2_ and EC values were also higher at 3 km/h than at higher speeds. This is further supported by the perceived exertion score (Borg Scale), where subjects reported greater perceived exertion in the perturbed compared to the flat condition at 3 km/h (+ 58%). These results indicate that the 3 km/h speed was uncomfortable because it was too slow compared to the subjects’ usual walking speed, and unexpected perturbations exacerbated this difference.

The difference in RPE between perturbed and flat conditions was significant at 5 km/h (+ 16%) but not at 4 km/h. Since there were no differences between conditions in terms of metabolic and ventilatory variables (VO_2_, EC, and VE), the results suggest that at 4 km/h, participants were in a “comfort zone” where the speed was comfortable and manageable for them, allowing them to efficiently make the necessary postural/muscular adjustments required by the perturbations. This is elegantly supported by Saibene and Minetti^37 ^who showed that 1.1 m/s (4 km/h) is considered the optimal walking speed. The difference in perception at the higher speed could be caused by processes acting at a level different from the metabolic one.

Continuous exposure to unexpected perturbations is capable of challenging the system as it requires the use of alternative motor control strategies at the neuromuscular level. In fact, continuously variable and unexpected perturbations would exclude any short-term predictive behaviour of the system^35^. A previous study^38^ evaluated the EC on uneven surfaces during running, reporting an EC increase of + 5%. This increase was accompanied by changes in the variability of step width (+ 27%), length (+ 26%), and height (+ 125%) on uneven terrain. Additionally, step period variability on uneven terrain increased significantly by 30%^38^. Notable increases in mean muscle activity were observed in the vastus medialis (+ 7%), rectus femoris (+ 20%), and medial hamstring (+ 19%) muscles. However, no significant differences in mean muscle activity were observed considering the vastus lateralis muscle or any other lower-limb muscle between the conditions. Furthermore, the authors demonstrated a notable degree of EMG variability between conditions for the soleus and gastrocnemius (both + 14%), the vastus medialis (+ 15%), the rectus femoris (+ 35%), and the medial hamstring (+ 26%) muscles. An alternative approach to the previous study, which used disturbances created by the treadmill surface by placing blocks of different heights on the belt, was to examine the effects of unpredictable medio-lateral disturbances on a cohort of well-trained runners from a metabolic and electromyographic perspective at different speeds^36^. The authors showed that the cardio-metabolic parameters remained unaltered by the perturbations. Additionally, the amplitude and width of EMG activation peaks demonstrated no variation between conditions. However, the variability of EMG exhibited a notable impact from the conditions, exhibiting discrepancies in the coefficient of variation in peak amplitude and peak width, which were elevated in the perturbed compared to the flat condition. It can be reasonably deduced that the absence of notable cardio-metabolic variations between the conditions can be attributed to the limited contact times, which did not permit the subject to discern the alterations introduced by the treadmill and, consequently, to regulate an optimal metabolic response. This is corroborated by Voloshina’s^13,38^ studies on the impact of surface conditions on running and walking. Their findings indicate that the influence of surface irregularities on running performance is approximately 5%, whereas it is 27% for walking. However, the observed differences in the coefficient of variation in peak amplitude and peak width demonstrate how variations in medio-lateral inclination are detected by the neuromuscular system and addressed with different control strategies by the CNS^36^. Indeed, during walking, in the presence of perturbations in the medio-lateral direction, participants do not change their speed as a coping mechanism for stability, but rather they change the spatio-temporal parameters of their gait. Indeed, as demonstrated by Hak et al.^39,40 ^subjects did not change their walking speed in response to the balance perturbations, but their steps became shorter, faster, and wider as the intensity of the perturbations increased. With regard to the EC assessed under both even and uneven terrain conditions during treadmill walking, an increase of + 27–28% was observed in the latter^13,38^. These changes were accompanied by alterations in gait biomechanics, including a 4% reduction in step length, a 22% increase in step length variability, and a 36% increase in step width variability^13^. In support of this, a study by Grimmitt and colleagues^41^ found that a 1% increase in stride length variability was associated with a 0.7% increase in EC. Reducing the energy expenditure associated with unstable locomotion may not be the primary objective during movement. Sometimes, compromises may be necessary to prioritize other factors, such as safety or perceived comfort^35^.

From a motor control perspective, our results showed that four muscle synergies were required to accurately model the neuromuscular control during both stable and perturbed walking. These findings align with previous literature on gait synergies, which suggests that four to five muscle synergies (depending on the type and number of muscles acquired) are necessary to effectively describe human locomotion^28,32,42^. Results revealed that perturbed walking does not affect the motor control capacity (i.e., number of muscle synergies) of elderly participants, substantiating the hypothesis behind the muscle synergy theory, which states that a consistent set of muscle synergies can be used to describe slightly different motor tasks, such as flat and perturbed walking. In other words, the CNS seems to be able to modulate the existing set of muscle synergies to adapt to varying locomotion conditions. This conclusion is further supported by prior studies demonstrating a consistent number of muscle synergies across different walking conditions^32,35,43,44^. A reduction in the number of muscle synergies between stable and perturbed walking was observed only in studies that applied more intense walking perturbations capable of inducing falls^45,46^. The recruitment of a common set of muscle synergies for executing different motor tasks is also reported as a strategy for the central nervous system to respond more rapidly to force perturbations rather than generating a new set of muscle synergies^47^. These findings were further reinforced by a comprehensive analysis of the muscle synergies using the Full-Width at Half Maximum (FWHM), Weight Sparsity (WS), and Cosine Similarity (CS) parameters. Results revealed that, while the total number of gait synergies remained unchanged, the transition from stable to perturbed walking led to statistically significant modifications in gait synergy modulation. Specifically, the neural activation patterns (i.e., activation coefficients) were notably altered, as indicated by a significant increase in FWHM (*p* = 0.01), reflecting a broader and more prolonged activation of muscle synergies throughout the gait cycle. Even if participants’ stability state was not directly measured in our experimental protocol, we can assume that these unpredictable perturbations reduced participants’ stability compared to stable walking. Thus, it can be speculated that the temporal rearrangement (i.e., widening) of the neural activation patterns can represent an enhancement in the motor control’s resilience to cope with external disturbances. In other words, these findings may suggest a transition from an accurate to a more robust locomotor control to deal with continuously variable perturbations. These findings are supported by the study by Brüll et al.^33 ^which investigated the impact of gait perturbations on the spatiotemporal modulation of muscle synergies. Their findings indicated that the four basic gait synergies were preserved across all applied perturbations (including medio-lateral and antero-posterior, both unpredictable and predictable treadmill-induced perturbations). However, the temporal recruitment patterns within each synergy (activation coefficients) were strongly modified. The widening of activation coefficients during challenging walking tasks appears to be a common adaptive mechanism, as it has been observed in both healthy individuals^34^ and patients suffering from multiple sclerosis^48,49^. Furthermore, the accurate-to-robust transition appears to be influenced by walking speed. Specifically, the increase in FWHM (see Table 2) from stable to perturbed walking was more pronounced at 3 km/h (+ 15%) compared to 5 km/h (+ 4%). This may be attributed to the shorter stance phase at higher speeds, which limits the time available for sensory feedback processing and motor control strategy adaptation. As a result, participants may have been less able to discern and compensate for perturbations introduced by the treadmill at faster speeds^13^.

From a spinal-level perspective, the analysis of the WS (i.e., the number of muscles actively enrolled within each muscle synergy, see Table 3) and CS (i.e., the degree of correlation between couple of weight vectors extracted during walking conditions, see Table 4) parameters revealed no significant differences between stable and perturbed walking, suggesting that unpredictable perturbations did not substantially alter muscle recruitment patterns at the spinal level. This finding further supports the idea that adjustments to motor control strategies in response to external disturbances mainly occur at the supraspinal level, with cortical and subcortical structures likely playing a dominant role in modulating locomotor control under variable conditions.

Previous studies have shown that walking on uneven terrain increases kinematic variability and perceived instability in older adults, especially those with lower mobility function, with greater step-to-step variability and reduced walking speed compared to younger individuals^50,51^. Moreover, an increase in task difficulty is accompanied by a corresponding rise in cortical activity. This augmented cerebral activity may encompass augmented activation within a specific region and/or the recruitment of supplementary brain regions. To this end, the capacity to control complex walking tasks may be constrained by limited cognitive resources, particularly within the prefrontal cortex, affecting the quality of movement^52^. The prefrontal cortex, which is primarily responsible for cognitive processes, has been observed to undergo age-related declines, which may subsequently result in further impairments to motor control during complex walking tasks^53^. Therefore, the observed neuromuscular and energetic adaptations may represent a broader age-related strategy with the aim of maintaining more conservative or compensatory motor control (assessed through FWHM) at the expense of energy expenditure.

Gait activity is determined by multiple factors, both latent and contextual, which act synergistically, influencing both metabolic efficiency and neuromuscular control. The present work is positioned in this perspective. To the best of the authors’ knowledge, this is the first study to simultaneously evaluate both metabolic and motor control adaptations through a multi-modal approach in response to unpredictable medio-lateral perturbations during walking. The modifications in motor control strategies, as identified through the muscle synergy analysis, align with the metabolic findings, revealing wider neural activation patterns and increased EC during perturbed compared to flat walking, respectively. These findings suggest that coping with continuous perturbations prompts a shift toward more robust locomotor control, which may lead to higher metabolic demand. This association highlights the potential trade-off between stability and efficiency, reinforcing the idea that neuromuscular adaptations aimed at enhancing resilience against perturbations may require greater energy expenditure.

### Limitations and future perspectives

In this study, we must consider the sample size as a limitation, as it should be considered that if the results did not reach statistical significance, it may have been due to the small sample size. The results presented here cannot be considered conclusive, as they should be further investigated on a larger-scale sample that includes, potentially, individuals with pathologies as well as both young and adult individuals. Moreover, we investigated eight muscles on a single limb. This setup is considered the minimum required to perform assessments using the muscle synergy technique. A larger number of analyzed muscles (e.g., spinal erectors) could potentially reveal additional modules with different behaviours during perturbed condition^54^. However, since no one has ever examined the mechanical–bioenergetic interaction during walking under unpredictable medio-lateral perturbations, one of the future directions should be to explore these aspects. In the future, the combination of biomechanical parameters, spatio-temporal gait parameters, metabolic data, and perceived exertion should be further investigated to better define the associations with the study of muscle synergies.

## Materials and methods

### Participants

A convenience sample of 16 healthy older adults (8 males and 8 females; age = 69 ± 5 years; weight = 68 ± 10 kg; height = 1.67 ± 0.08 m; BMI = 24.3 ± 2.7 kg/m^2^) took part in this study. Inclusion criteria were: (i) an age of 65–80 years, (ii) home dwelling, and (iii) the ability to walk independently on a treadmill for at least 20 min^35^. Exclusion criteria were any previous history of neuromuscular disorders or injury in the last six months. All the participants were informed about the testing procedure. They provided written informed consent before participating in this study, which was approved by the local Ethical Advisory Committee (University of Verona —approval no: 11.R1/2023) and performed in accordance with the Helsinki Declaration.

### Experimental design

The study comprised two visits interspersed by 1 week. Visit 1 consisted of familiarization and anthropometric measures (approximately 1 h), while Visit 2 was the testing session (approximately 2 h). During Visit 1, the participants were familiarized with the Rated Perceived Exertion scale (RPE, Scale: 0-100) and the unstable condition for each speed used during the test. At the second visit (Visit 2), participants were tested on a treadmill using two modalities (stable and unstable, presented in random order) and at three different speeds (3, 4, and 5 km/h)^37^, with the slope fixed at 1%^55^. During this session, metabolic data and EMG signals from 8 different muscles of the dominant lower limb were collected, as well as scores from the RPE scale.

### Data recordings

**Fig. 2:**
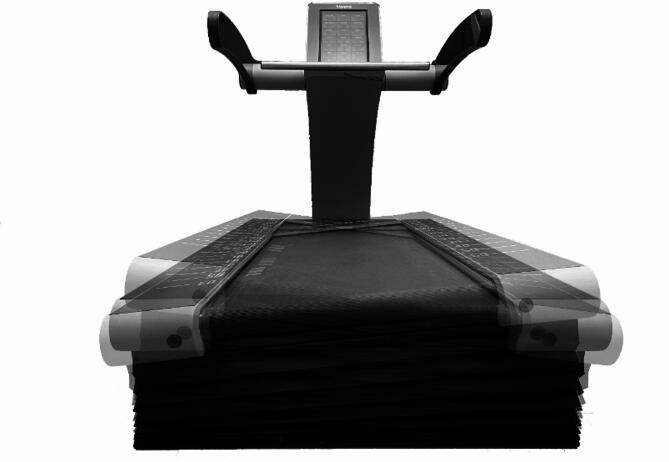
Sketch of the treadmill used with the medio-lateral oscillations of + 7/-7° highlighted. Figure created from original photographs and processed using Adobe Photoshop (version 21.1.1) for illustrative purposes.

During the second visit, participants completed a 6-minute trial for each walking speed (i.e., 3, 4, and 5 km/h), both in Flat Condition (FC) and Unpredictable Roll Variations (URV) condition, in randomized order. Each trial was separated by a two-minute rest period. During the perturbed condition, a randomized instability routine^36 ^previously used by Skroce and colleagues^33 ^was given based on the following criteria: (i) lateral inclination ranged from − 7° to + 7° and (ii) each position was maintained for 1 to 3 s. To make the results comparable, the same routine was administered to all subjects and at all speeds. All walking tests were conducted on a motorized treadmill (ReaxRun, Reaxing, Milan, Italy; see Fig. 2).

Metabolic data were recorded through a breath-by-breath analyzer (K5, Cosmed, Rome, Italy) that was previously set and calibrated according to the manufacturer’s instructions for each testing session. Lactate samples from the ear lobe were collected and analyzed (Biosen C-line, EKF Diagnostics, Barleben, Germany) before and after every trial.

Gait data were collected through five wireless probes (one used to acquire linear accelerations and four to record EMG signals; sampling frequency: 2048 Hz; gain: 200 V/V; bandwidth: 10–500 Hz; A/D converter resolution: 16 bit; DueLite, OTBioelettronica, Turin, Italy), which allowed the positioning of 8 pairs of bipolar electrodes (CDE-C, Ø=24 mm, OTBioelettronica, Turin, Italy). The electrodes were placed, according to the SENIAM guidelines^56 ^on the following 8 muscles of the right (dominant) lower limb: Bicep Femoris (BF), Gastrocnemius Medialis (GM), SemiTendinosus (ST), Peroneus Longus (PL), Soleus (SOL), Tibialis Anterior (TA), Vastus Lateralis (VL) and Vastus Medialis (VM). The inter-electrode distance was 20 mm. Before the placement of electrodes, the skin was prepared by shaving the hair, applying an abrasive paste (Nuprep, Weaver and Company, Aurora, CO, USA), and, in the end, cleaning with water to reduce the impedance^57^. To prevent movement artifacts, the electrodes and cables were secured to the subjects’ skin with adhesive tape (Hypafix, 5 × 10 cm; Leukoplast, Hull, UK). Moreover, one wireless probe (2048 Hz; DueLite, OTBioelettronica, Turin, Italy) was placed over the right tibia through adhesive tape to detect gait events through the accelerometer data.

At the beginning and end of each trial, subjects were asked to provide self-assessments regarding RPE.

### Data analysis - metabolic analysis

The variables recorded included oxygen consumption (VO_2_), minute Ventilation (VE), Heart Rate (HR), and blood Lactate concentration (La).

The net energy cost ($\:EC$) of constant walking speed^58^ was computed according to the following formula (1):

1 $$\:EC\:=\frac{{VO}_{2net}+\:ELab}{v}$$

where $\:{VO}_{2net}$ (expressed in mlO_2_·min^− 1^·kg^− 1^) represents the net oxygen uptake, and $\:v$ is the treadmill speed (expressed in m·min^− 1^). $\:ELab$ (ml·min^− 1^·kg^[− [1^) is regarded as the energy derived from anaerobic lactic energy sources. $\:ELab$ is conventionally determined by multiplying the net lactate production by the energy equivalent and dividing it by the total exercise duration (i.e. 6 min), expressed as (mM·ml·kg^− 1^·mM^− 1^)·min^−1^. Since during walking the subjects did not produce a lactate buildup as no anaerobic component was utilized, the $\:ELab$ (mlO_2_·min^− 1^·kg^−1^) was not considered in the $\:EC$ formula (1). Finally, the energy cost was expressed in J/kg/m using an energy equivalent which takes into account the respiratory exchange ratio (RER)^58^: VO_2_·(4.94·RER + 16.04) [J·mlO_2_ ^− 1^].

Since, at intensities within the moderate domain, the steady state is typically achieved within the first three minutes, an additional sub-analysis of the physiological variables was performed halfway through each step (EC_3min_ and EC_6min_). This analysis aimed to identify potential duration-dependent metabolic alterations that could arise due to prolonged exposure to both flat and perturbed conditions. Specifically, this approach was intended to investigate whether extended exercise duration would elicit changes in oxygen uptake, lactate accumulation, or other metabolic responses as the body adapted to the varying demands of each condition.

### Data analysis - muscle synergy extraction and analysis

Gait Cycles (GCs) were segmented based on the shank-mounted IMU recordings. The identification of Heel-Strike (HS) and Toe-Off (TO) events was based on angular velocity signals in the sagittal plane obtained from the gyroscope^59,60^. Specifically, an HS event was identified as the time instant corresponding to the minimum angular velocity occurring after the peak angular velocity of the shank, which corresponds to the mid-swing phase. A TO event, instead, was identified as the time instant corresponding to the minimum angular velocity occurring just before the peak angular velocity. To consider steady-state behaviour only, the first and the last 10 gait cycles of each task condition and speed were discarded. The walking task was divided into two time-windows (i.e., the first 3 min and the last 3 min of each trial) to assess potential duration-dependent neuromuscular alterations that could arise due to prolonged exposure to both flat and perturbed conditions^61^. This approach aligns with the methodology used in the Energy Cost analysis.

Before extracting muscle synergies, EMG data were pre-processed according to the following standard steps. Firstly, an envelope extraction was performed through a 4th -order high-pass Butterworth filter at 35 Hz, a full-wave rectification, and a 4th -order low-pass Butterworth filter at 10 Hz, according to Ghislieri and collegues^62,63^. Secondly, each GC was time-normalized to 1000 samples^64 ^assigning 600 samples to the stance phase and 400 samples to the swing phase. This was done to avoid biases due to the absolute durations of gait sub-phases and to help assess the temporal contribution of the different muscle synergies to each gait cycle sub-phase^65^. Then, an amplitude normalization was performed to the global maximum of each acquired muscle^66^. Lastly, the Non-Negative Matrix Factorization (NNMF) was applied to the time- and amplitude-normalized EMG envelopes following the optimized version proposed by Lee and Seung^67^.

The NNMF algorithm enables the decomposition of the original data (M) into two low-dimensional components: (i) the activation coefficients (C), which describe the temporal modulation of motor control (i.e., the timing of muscle group activation over task duration) and (ii) the weight vectors (W), which capture the spatial information of motor control (i.e., the muscles synergistically activated). The MATLAB function “*nnmf*” (release R2023b; MathWorks Inc., Natick, USA) was used to extract the “time-invariant” muscle synergy model from the EMG data, setting the maximum number of iterations equal to 1000, the number of replicates equal to 15, and the function and search tolerance equal to 1e^− 6^^62,63^. A sparseness constraint was applied to the first initialization of the weight vector to improve algorithm performance^68^. The NNMF algorithm was applied to the same EMG data changing the muscle synergy number (N) between 1 and 8 (i.e., the minimum and maximum number of recorded muscles in the study) to explore different factorization solutions. To assess the goodness of the muscle synergy model in describing the original EMG data, the R^2^ similarity between the original and the reconstructed EMG signals was computed for each tested number of synergies. Before muscle synergy analysis, NNMF solutions extracted from different walking conditions and subjects were sorted in the same order according to Pearson’s correlation coefficients calculated between each pair of weight vectors. The activation coefficients were then sorted consequently.

For each volunteer, each walking condition, and both time-windows, muscle synergies were quantitatively analyzed based on: (i) the required number of muscle synergies (N_syn_), (ii) the full width at half maximum (FWHM) of the activation coefficients, and (iii) the sparsity of the weight vectors (WS). Additionally, muscle synergy compositions were qualitatively compared.

*Number of muscle synergies (N*_*syn*_*).* To avoid setting arbitrary cut-off thresholds on model reconstruction quality, the required number of muscle synergies for modelling the original EMG data was determined using the “elbow” criterion - identifying the point of highest curvature on the R^2^ vs. number of muscle synergies curve. Specifically, the curvature was calculated for each set of three consecutive points along the R^2^ vs. number of muscle synergies curve, iteratively excluding the smallest number of synergies^27^. The optimal number of muscle synergies to accurately model the walking task across the entire sample population was then defined as the mode of the synergy numbers calculated for each volunteer. N_syn_ ranges from 1 to 8, corresponding to the minimum and maximum number of muscles recorded in the study.

*Full Width at Half Maximum (FWHM).* The duration of each activation coefficient was computed cycle-by-cycle as the number of time instants exceeding half of the signal’s maximum value (i.e., full width at half maximum), after subtracting the minimum within the same gait cycle. The FWHM values for each participant were then averaged across gait cycles and muscle synergies, obtaining a single representative FWHM value per individual^62,63^. FWHM is expressed in percentage of the gait cycle duration, ranging from 0% (indicating a null activation duration) to 100% (indicating a long activation duration).

*Weight vector Sparsity (WS)*. WS was defined as the number of significantly active muscles per muscle synergy. To determine this, an adaptive threshold ($\:t{h}_{k}$) was computed for each muscle ($\:k\:$) as follows (2):

2 $$\:t{h}_{k}=\frac{\text{min}\left({W}_{k}\right)}{\text{max}\left({W}_{k}\right)}$$

where $\:{W}_{k}$ is a vector containing the contributions of muscle $\:k\:$ to each muscle synergy. The overall threshold ($\:th\:$) was then determined as the maximum of all individual $\:t{h}_{k}$ values. A muscle was then considered significantly active in a given synergy if its contribution (i.e., weight vector value) exceeded $\:th$. WS ranges from 1 to 8, reflecting the minimum and maximum number of recorded muscles in this study.

*Cosine Similarity (CS)*. To quantify the degree of correlation between couple of weight vectors extracted during different walking conditions, the Cosine Similarity (CS) was computed for each muscle synergy^69^. The CS between two weight vectors was defined as the normalized scalar product between the vectors. CS values range between 0 (indicating a null correlation between two weight vectors) and 1 (indicating a strong correlation between two weight vectors).

### Statistical analysis

To assess data distribution normality, the Shapiro-Wilk test was performed. In the case of normally distributed data, a 2-way ANOVA (Speed × Condition) and a 3-way ANOVA (Speed × Condition × Time Window) were performed to test statistically significant differences in metabolic and muscle synergy data, respectively. Otherwise, the Friedman test for non-parametric data was performed. *Post-hoc* tests were corrected with Bonferroni method for multiple comparisons. The effect size in ANOVAs was reported as $\:{\eta\:}_{p}^{2}$. Threshold values^70^ for $\:{\eta\:}_{p}^{2}$ were set at 0.01, 0.06, and 0.14, corresponding to small, medium, and large effect sizes, respectively. Sphericity was assessed using Mauchly’s test. In cases where the assumption of sphericity was violated (*p* < 0.05), degrees of freedom were corrected using the Greenhouse-Geisser adjustment to reduce the risk of Type I error. For all the statistical analyses, the significance level (α) was set equal to 0.05. The outliers were manually removed. All the statistical analyses were performed in JASP (Version 0.19).

## Electronic supplementary material

Below is the link to the electronic supplementary material.

Supplementary Material 1

Supplementary Material 2

Supplementary Material 3

## Data Availability

All data are available from the corresponding author upon reasonable request.

## Abbreviations

BF: Bicep femoris
CNS: Central nervous system
CS: Cosine similarity
EC: Energetic cost
EC_3min_: Energetic cost assessed from zero to 3 min from the onset
EC_6min_: Energetic cost assessed from 3 min to 6 min
ELab: Energy derived from anaerobic lactic energy sources
FC: Flat condition
FWHM: Full width at half maximum
GC: Gait cycle
GM: Gastrocnemius medialis
HR: Heart rate
HS: Heel-strike
La: Blood lactate concentration
NNMF: Non-negative matrix factorization
PL: Peroneus longus
RER: Respiratory exchange ratio
SOL: Soleus
ST: SemiTendinosus
TA: Tibialis anterior
TO: Toe-off
URV: Unpredictable roll variations
VAF: Variance accounted for
VE: Minute ventilation
VL: Vastus lateralis
VM: Vastus medialis
VO_2_: Oxygen consumption
WS: Weight sparsity
