# Supplementary material for: Muscle synergies and metabolic adaptations during perturbed walking in older adults
Source: Sci Rep. 2025 Jul 2;15:23597. doi: 10.1038/s41598-025-07835-4 (PMC12223200; doi:10.1038/s41598-025-07835-4)
Supplement: Supplementary file 1 — Supplementary Material 1 [file 41598_2025_7835_MOESM1_ESM.docx]

**Supplementary Materials**


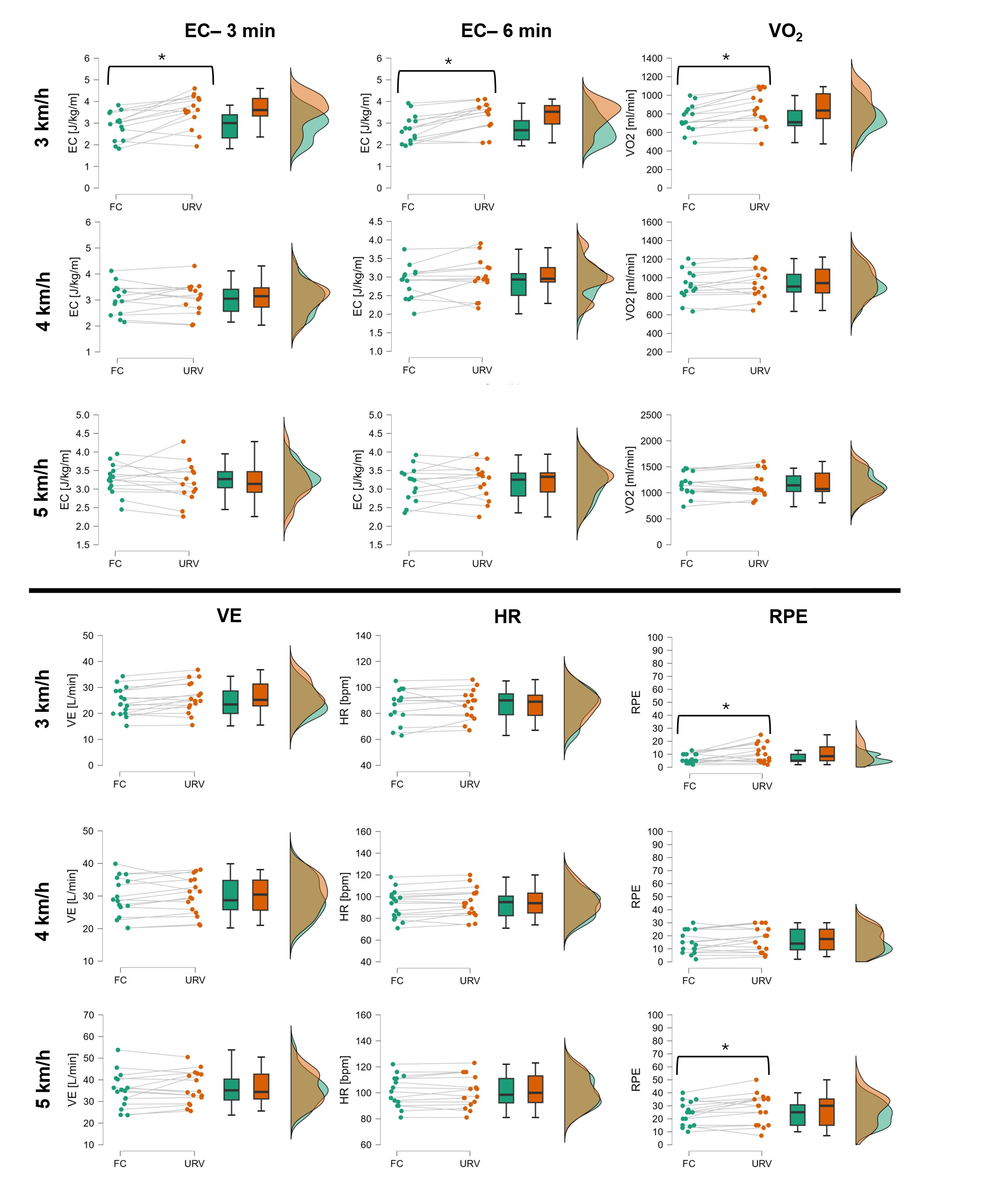


**Figure 1S**. Raincloud plots of the metabolic and exertion variables were analyzed in this study. For each parameter and condition, the raincloud plot shows, sequentially, the raw jittered data points of each individual, the central tendency through a boxplot, and the data distribution through a split-half violin plot. ^*^*p* ≤ .05.


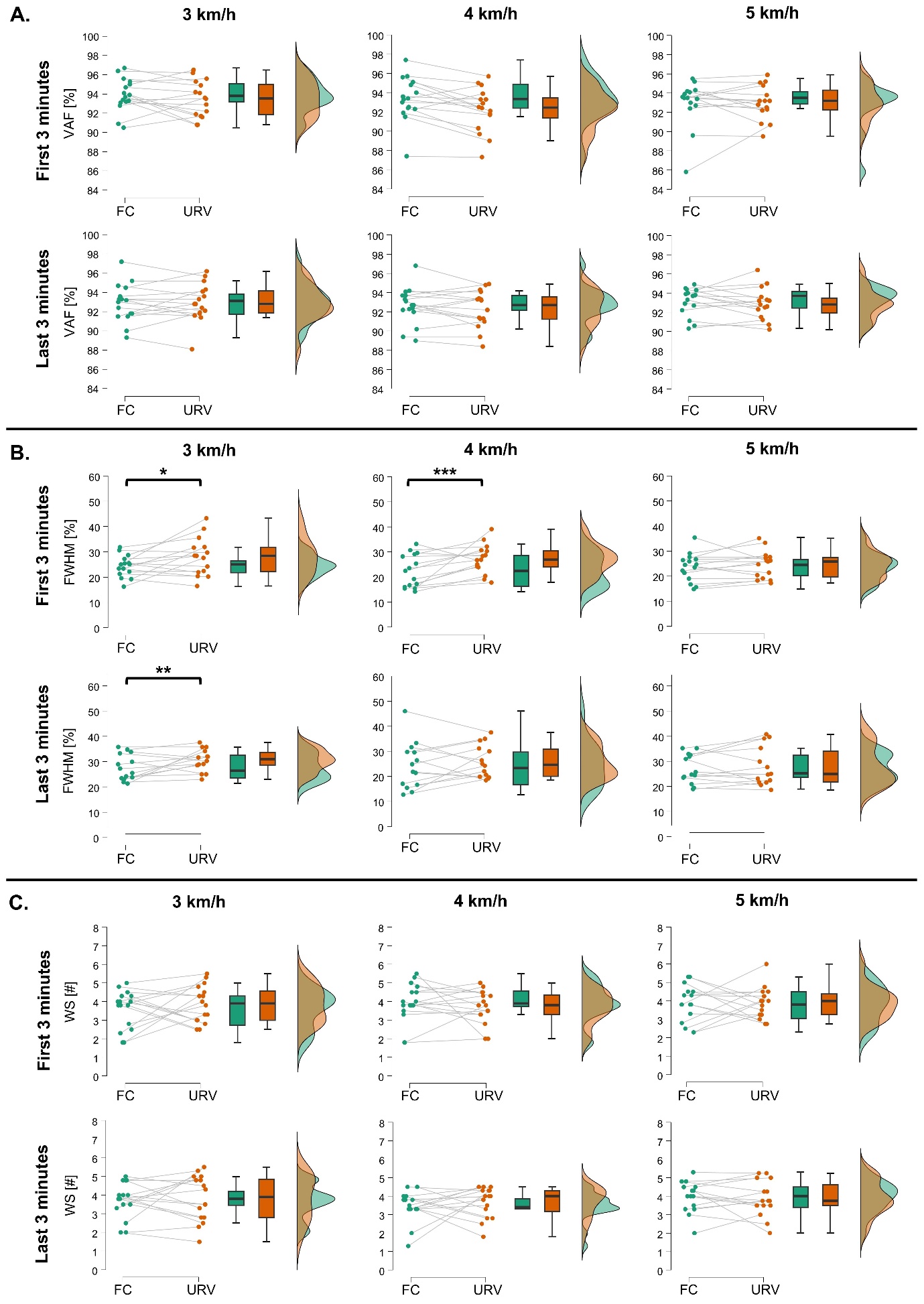


**Figure 2S**. Raincloud plots of muscle synergy-derived parameters, grouped by walking speed and time windows (first vs. last 3 minutes). Panel A, Panel B, and Panel C illustrate the data distribution for VAF, FWHM, and WS parameters, respectively. Statistical significance: **p* ≤ .05, ***p* ≤ .01, ****p* ≤ .001.
