# Supplementary figures and images for: Muscle synergies and metabolic adaptations during perturbed walking in older adults
Source: Sci Rep. 2025 Jul 2;15:23597. doi: 10.1038/s41598-025-07835-4 (PMC12223200; doi:10.1038/s41598-025-07835-4)

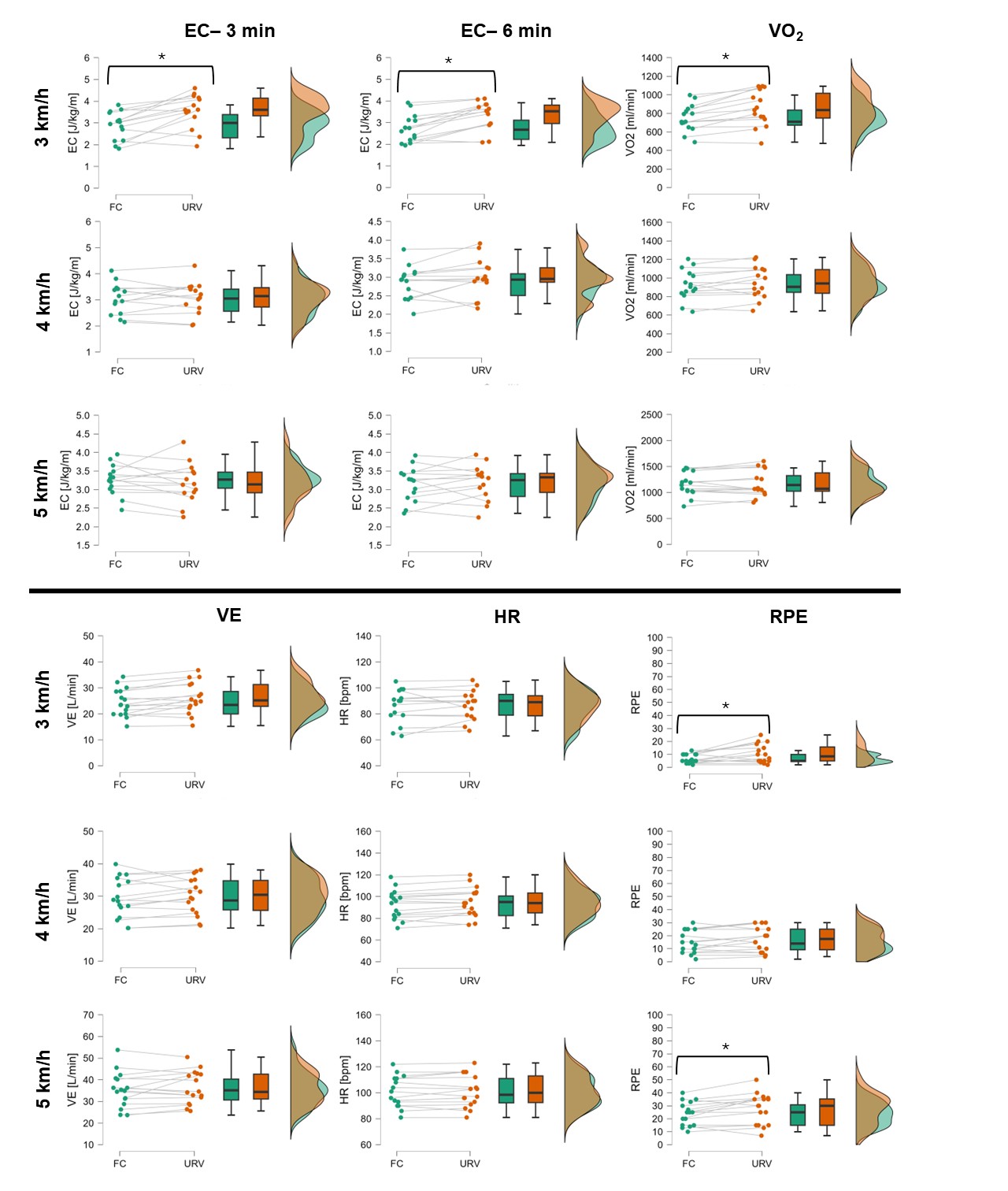

Supplement: Supplementary file 2 — Supplementary Material 2 [file 41598_2025_7835_MOESM2_ESM.jpg]

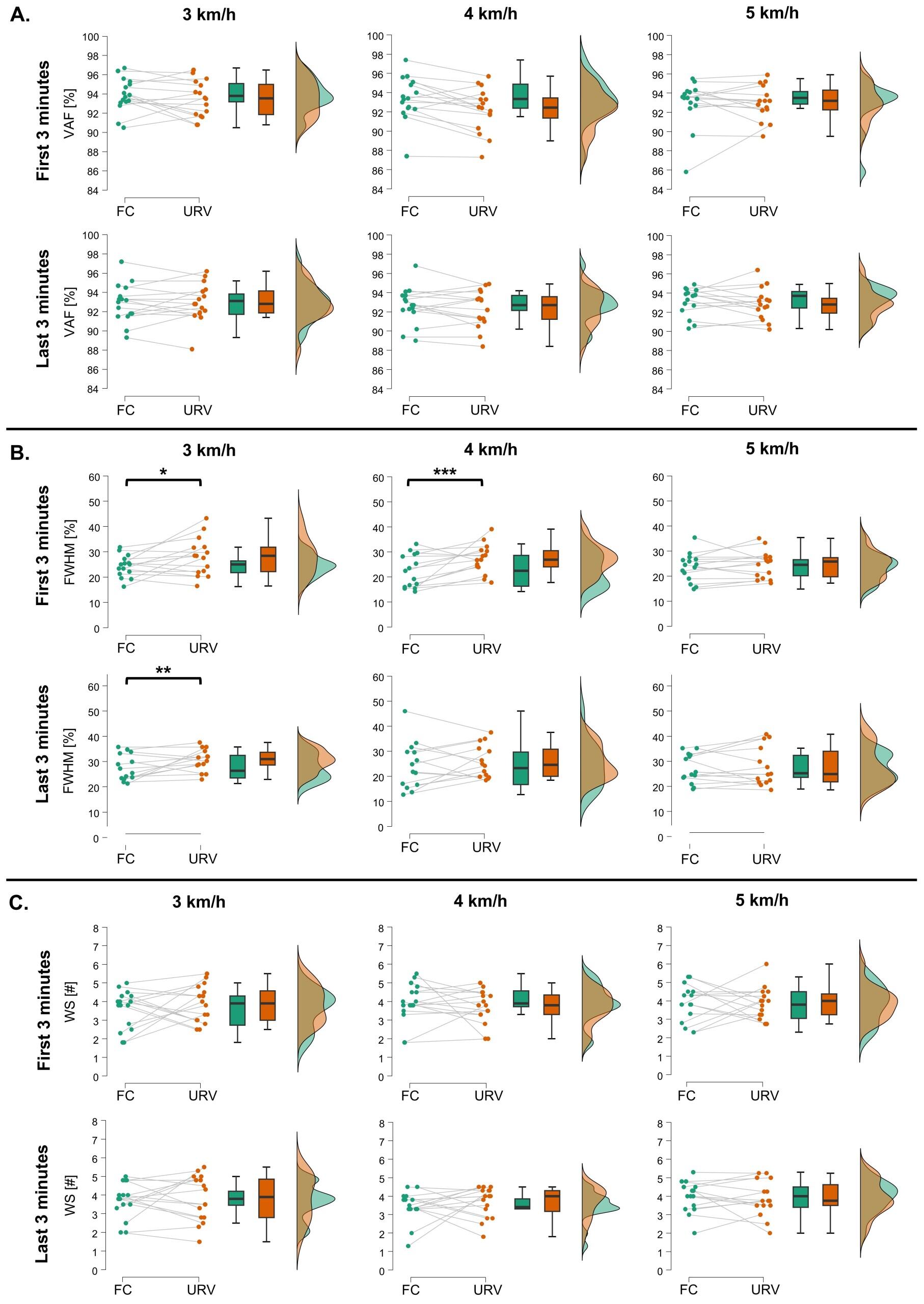

Supplement: Supplementary file 3 — Supplementary Material 3 [file 41598_2025_7835_MOESM3_ESM.jpg]
